# Selection of Specific Peptides for *Coccidioides* spp. Obtained from Antigenic Fractions through SDS-PAGE and Western Blot Methods by the Recognition of Sera from Patients with Coccidioidomycosis

**DOI:** 10.3390/molecules23123145

**Published:** 2018-11-30

**Authors:** Esperanza Duarte Escalante, María Guadalupe Frías De León, Luz Gisela Martínez García, Jorge Herrera, Gustavo Acosta Altamirano, Carlos Cabello, Gabriel Palma, María del Rocío Reyes Montes

**Affiliations:** 1 Departamento de Microbiología y Parasitología, Facultad de Medicina, Universidad Nacional Autónoma de México (UNAM), Ciudad Universitaria No. 3000, México Cd. Mx. 04510, Mexico; dupe@unam.mx (E.D.E.); marga299@yahoo.com.mx (L.G.M.G.); 2 Hospital Regional de Alta Especialidad de Ixtapaluca, Carretera Federal México—Puebla Km. 34.5, Pueblo de Zoquiapan, Ixtapaluca 56530, Mexico; magpefrias@gmail.com (M.G.F.D.L.); mq9903@live.com.mx (G.A.A.); 3 Unidad de Servicios de Apoyo a la Investigación y la Industria (USAII), Facultad de Química, Universidad Nacional Autónoma de México (UNAM), Ciudad Universitaria No. 3000, México Cd. Mx. 04510, Mexico; jherredmx@yahoo.com.mx; 4 Instituto Nacional de Enfermedades Respiratorias (INER), Calzada de Tlalpan 4502, Belisario Domínguez Sección XVI, Tlalpan, México Cd. Mx 14080, Mexico; carloscginer@gmail.com (C.C.); gabpal52@yahoo.com.mx (G.P.)

**Keywords:** coccidioidomycosis, immunodiagnosis, peptides, SDS-PAGE, *Coccidioides* spp.

## Abstract

Antigenic fractions of 100, 50, 37, and 28 kDa obtained through the SDS-PAGE method that were more frequently recognized by anti-*Coccidioides* antibodies in the sera of coccidioidomycosis patients were selected using western blotting. Subsequently, these bands were sequenced, and the obtained proteins were analysed by BLAST to choose peptides specific for *Coccidioides* spp. from among the shared aligned sequences of related fungi. A peptide specific for *C. immitis* was selected from the “GPI anchored serine-threonine rich protein OS *C. immitis*”, while from the “uncharacterized protein of *C. immitis*”, we selected a peptide for *C. immitis* and *C. posadasii*. These proteins arose from the 100 kDa antigenic fraction. From the protein “fatty acid amide hydrolase 1 of *C. posadasii*” that was identified from the 50 kDa antigenic fraction, a peptide was selected that recognized *C. immitis* and *C. posadasii*. In addition, the analysis of all the peptides (353) of each of the assembled proteins showed that only 35 had 100% identity with proteins of *C. immitis* and *C. posadasii*, one had 100% identity with only *C. immitis*, and one had 100% identity with only *C. posadasii.* These peptides can be used as diagnostic reagents, vaccines, and antifungals.

## 1. Introduction

Coccidioidomycosis or San Joaquin valley fever is a disease caused by fungi of the genus *Coccidioides*, in which two species with almost identical phenotypes, *C. immitis* and *C. posadasii*, are recognized [1]. This disease is one of the most important endemic mycoses in North America. However, there are also significant endemic areas in other countries, such as Guatemala, Honduras, Venezuela, Colombia, Brazil, and Argentina. In these countries, coccidioidomycosis is not a disease with mandatory reporting; therefore, epidemiological data regarding this mycosis are still scarce, and investigations are restricted to retrospective studies and to the publication of clinical cases [2,3,4,5,6,7,8,9,10,11,12,13,14,15,16]. Therefore, the actual incidence of this disease is unknown.

The clinical manifestations of coccidioidomycosis can be confused with those of other nosological entities, which hinders its diagnosis. Traditionally, the diagnosis of coccidioidomycosis is made considering clinical and radiological results and laboratory analyses. The conventional diagnosis for coccidioidomycosis has been based on fungal identification via direct examination using freshly stained samples or different staining techniques and by the culture of tissue or fluid samples. However, the retrieval rate of this pathogen through culture varies from 0.4% in blood to 8.3% in respiratory tract samples [17]. The use of immunodiagnostic methods is still recommended, such as immunodiffusion (ID), complement fixation (CF), and tube precipitation (TP). These methods traditionally use coccidioidin, a crude extract formed from a large number of fungal components that favours cross-reactivity between the antibodies of patients with different fungal (*Blastomyces dermatitides*, *Histoplasma capsulatum*) and bacterial infections [18]. Lindsley et al. [19] showed that coccidioidin detects IgG antibodies, but when coccidioidin is treated with heat, it detects IgM antibodies. Therefore, the immunodiffusion-complement fixation (IDCF) sensitivity is 77%, while the immunodiffusion-tube precipitation (IDTP) sensitivity is between 75% and 91% [20]. These findings led to the production of pure antigens with well-defined physicochemical properties for the diagnosis of coccidioidomycosis, such as the coccidioidal CF and IDCF antigen, corresponding to a chitinase. The gene encoding this antigen was cloned, and a functional recombinant antigen called 47 kDa CF-chitinase (rCF) was obtained [21]. This antigen has shown a sensitivity of 96.9% and a specificity of 100% in an enzyme-linked immunosorbent assay (ELISA) [22]. However, it shares an epitope with *H. capsulatum* and *B. dermatitidis* [23]. The proline-rich antigen (Ag2/PRA), or antigen 2, is another specific antigen for *Coccidioides* spp., obtained by the deglycosylation of a spherule extract. This antigen has been favourably evaluated (in mice) as a vaccine candidate [24]. The gene encoding this antigen has been cloned and the recombinant antigen (rPRA) obtained, which recognizes IgG antibodies from patients with progressive pulmonary or extrapulmonary coccidioidomycosis. It has a high sensitivity (88%) and specificity (97%) for the diagnosis of coccidioidomycosis [25]. Filho et al. [26] evaluated the reactivity of a local antigen that was extracted from a strain of *C. posadasii*. This antigen was extracted with ammonium sulfate and was characterized by 1-D electrophoresis, showing good specificity and reactivity by radial immunodiffusion and western blot. Two well-defined bands were observed, with one corresponding to β-glucosidase and the other to glutamine synthetase; therefore, the authors proposed them to be a rapid diagnostic tool with a low cost and without the risk of the direct manipulation of the microorganism. Likewise, De Aguiar Cordeiro et al. [27] described the preparation of two antigens (F0-90 and F60-90) for the detection of human anti-*Coccidioides* antibodies by ID and EIA (enzyme immunoassay). The antigens were tested against serum samples from patients with coccidioidomycosis, histoplasmosis, and paracoccidioidomycosis, as well as from healthy individuals. The highest reactivity in the ID tests was observed with the F0-90 antigen, while the ELISA showed the best results with the F60-90 antigen. The F0-90 antigen showed cross-reactivity with *Paracoccidioides*, whereas F60-90 had cross-reactivity with *Paracoccidioides* and *Histoplasma*. Therefore, the authors suggested the commercial use of the F0-90 and F60-90 antigens for the presumptive diagnosis of coccidioidomycosis by ID or EIA, respectively.

Due to the disadvantages of cross-immunity, several researchers have oriented their work towards obtaining biomarkers with specificity and immunodominance properties, as in the case of Navalkar et al. [28], who applied the immunosignature technique. These authors used two peptide types to classify coccidioidomycosis: (1) a peptide set created by tiling four of the most immunodominant proteins of *C. immitis* to create 83 “epitope peptides” so that these peptides interact specifically with anti-*Coccidioides* antibodies; and (2) a microarray chip containing 10,000 printed, randomly sequenced peptides, which serve as artificial *Coccidioides* antigens to randomly interact with patient antibodies. The authors showed that, compared with the epitope peptides, the randomly sequenced peptides had a greater precision when classifying the different stages of coccidioidomycosis. Currently, there is a greater interest in molecular mimicry, particularly in using low molecular weight peptides, which have generated considerable expectations as substitutes for natural antigens [29]. Therefore, this work aims to select specific peptides for *Coccidioides* spp. that are obtained from antigenic fractions through SDS-PAGE and western blot by the recognition of sera from patients with coccidioidomycosis.

## 2. Results

### 2.1. Species Identification for Isolates of Coccidioides spp.

Six isolates were identified as *C. posadasii* (HU-1, 073129, 083376, GGM, ZVJ, QR) and five as *C. immitis* (M40-05, PRA, 30.1, 43.3, 62).

### 2.2. Coccidioidins Characterization

**Figure 1:**
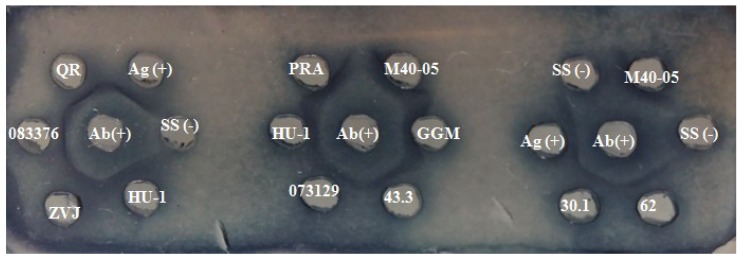
Immunological identification of coccidioidins. The identification of coccidioidins, based on the appearance of identity bands compared to the reference antigen through ID, was carried out according to the Methods section. Ag (+): Reference antigen; Ab (+): anti-*Coccidioides*-positive serum; SS (-): Saline solution.

The coccidioidins obtained from the isolates of *C. immitis* and *C. posadasii* included in this study revealed antigenic identity by ID, and all showed double identity bands (Figure 1).

### 2.3. Electrophoresis (SDS-PAGE)

**Figure 2:**
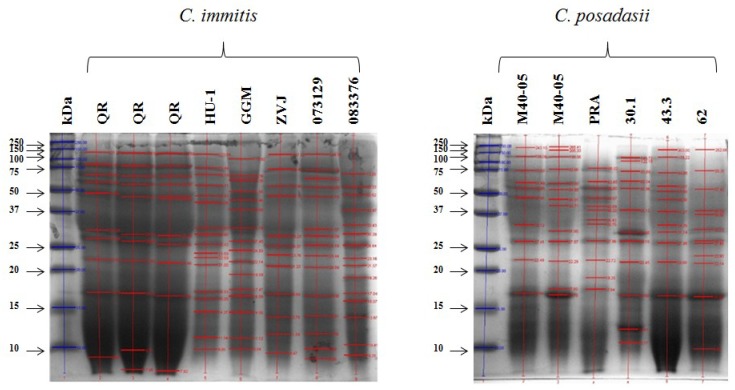
Electrophoresis (SDS-PAGE) of coccidioidins of *C. immitis* and *C. posadasii*. The molecular weight of each band of the protein pattern was analysed with Quantity One^®^ 1-D Analysis software (Bio-Rad). A concentration of 12% polyacrylamide was used. M: Molecular size marker (250 kDa).

For the SDS-PAGE assays, the protein concentration was adjusted to 3.3 mg/mL for all samples. Despite using different polyacrylamide concentrations in the SDS-PAGE gels (7.5, 10, and 12%), it was not possible to identify species-specific bands. However, when using 12% gels, bands with a wide range of molecular weights (10 to 250 kDa) were observed for exoantigens of *C. immitis* and *C. posadasii* (Figure 2). Table 1 shows the binary data matrix for the presence/absence of bands identified in the electrophoretic patterns for the evaluated coccidioidins (Figure 2).

### 2.4. Reactivity of Antigenic Fractions by WB

To optimise the antigen and antibody concentrations in the WB assays, the antigenic fractions were tested via dot blot using 11 serum samples that recognized *C. immitis* and *C. posadasii* antigens. The optimal antigen concentration was 0.66 mg/mL; the dilution of the primary antibody was 1/250, and the dilution of the secondary antibody was 1/5000. To demonstrate the recognition of antigenic fractions with 27 anti-*Coccidioides* sera samples obtained from patients, they were only tested with one coccidioidin of *C. immitis* (M40-05) and one of *C. posadasii* (HU-1). From the presence/absence data matrix (Supplementary Material Table S1), four bands were chosen for sequencing, according to the highest recognition frequency with each of the tested sera: 100, 50, 37, and 28 kDa (Table 2).

### 2.5. Analysis of Assembled Sequences

We identified eight proteins (assembled sequences) corresponding to the 100 kDa band, 14 proteins (assembled sequences) corresponding to the 50 kDa band, 14 proteins (assembled sequences) corresponding to the 37 kDa band, and 13 proteins (assembled sequences) corresponding to the 28 kDa band. For the sequences of proteins corresponding to the 100 kDa band, the analysis performed with the BLAST program showed two sequences: one as “GPI anchored serine-threonine rich protein OS *C. immitis*”, with access number A0A0J6YAG6_COCIT; and the other as “uncharacterized protein OS *C. immitis*”, with access number A0A0J8R1I4_COCIT. From the 50 kDa band, a sequence was identified as “fatty acid amide hydrolase 1 OS *C. posadasii*”, with access number A0A0J6F383_COCPO. From the 37 and 28 kDa bands, no sequence was identified with the conditions proposed for selection (Table 3).

**Figure 3:**
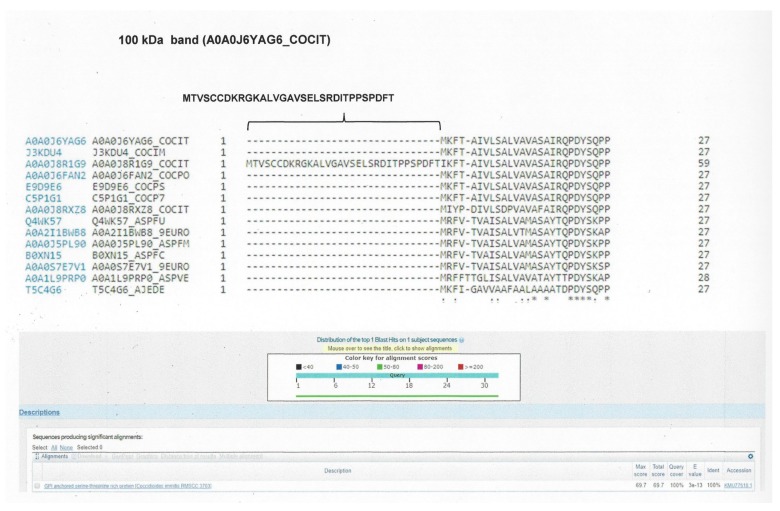
Alignment map with the BLAST protein program. The selected peptide of the assembled protein corresponding to the 100 kDa band is shown, identified through aligned sequences of *Coccidioides* spp. and related fungi. The selected peptide was compared through the BLAST program to confirm its specificity.

**Figure 4:**
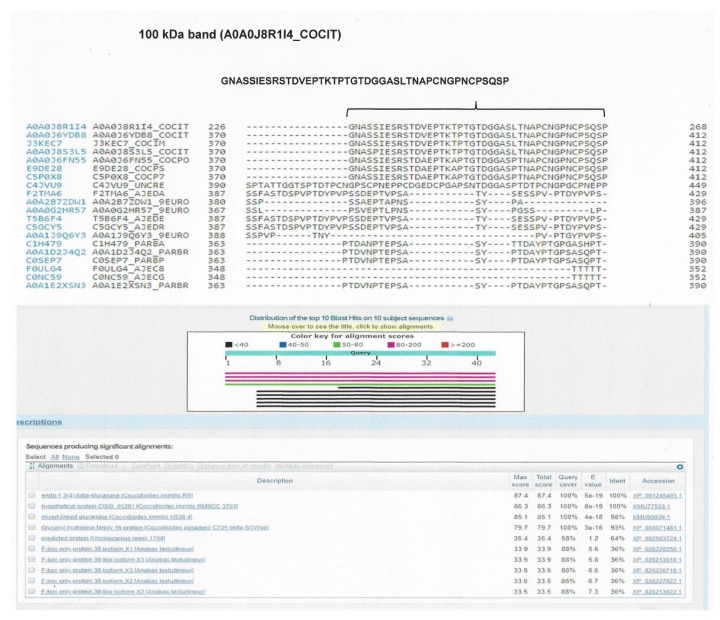
Alignment map with the BLAST protein program. The selected peptide of the assembled protein corresponding to the 100 kDa band is shown, identified through aligned sequences of *Coccidioides* spp. and related fungi. The selected peptide was compared through the BLAST program to confirm its specificity.

**Figure 5:**
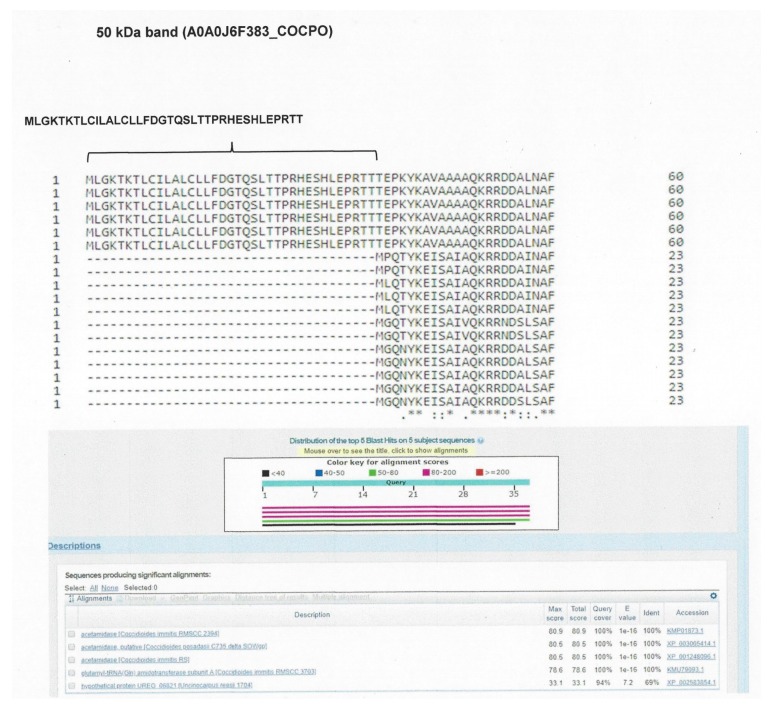
Alignment map with the BLAST protein program. The selected peptide of the assembled protein corresponding to the 50 kDa band is shown, identified through aligned sequences of *Coccidioides* spp. and related fungi. The selected peptide was compared through the BLAST program to confirm its specificity.

From the alignment result of the protein “GPI anchored serine-threonine rich membrane of *C. immitis*” with the related fungi, the peptide MTVSCCDKRGKALVGAVSELSRDITPPSPDFT was selected, which was specific for *C. immitis*. From the “uncharacterized protein of *C. immitis*”, with access number A0A0J8R1I4_COCIT, peptide GNASSIESRSTDVEPTKTPTGTDGGASLTNAPCNGPNCPSQSP was selected, which was present in both *C. immitis* and *C. posadasii*. Likewise, from the alignment of the protein “fatty acid amide hydrolase 1 of *C. posadasii*”, with access number A0A0J6F383_COCPO, the peptide MLGKTKTLCILALCLLFDGTQSLTTPRHESHLEPRTT was selected, which was present in *C. immitis* and *C. posadasii* (Figure 3, Figure 4 and Figure 5). To confirm the specificity of the selected peptides, they were analysed using the BLAST protein program.

### 2.6. Peptide Analysis

The analysis of all the peptides from each assembled protein showed that out of the 91 identified peptides corresponding to the 100 kDa band, nine had 100% identity with *C. immitis* and *C. posadasii* (Supplementary Material, Table S2). For the 50 kDa band, 50 peptides were identified; four showed 100% identity with *C. immitis* and *C. posadasii* and one showed 100% identity with only *C. posadasii* (Supplementary Material, Table S3). For the 37 kDa band, 152 peptides were identified; eight showed 100% identity with *C. immitis* and *C. posadasii*, and one showed 100% identity with only *C. immitis* (Supplementary Material, Table S4). For the 28 kDa band, 60 peptides were identified; 14 showed 100% identity with *C. immitis* and *C. posadasii*, and one showed 100% identity with only *C. posadasii* (Supplementary Material, Table S5).

## 3. Discussion

The diagnosis of coccidioidomycosis can be difficult in patients with acute and fulminant disease, especially if they are immunosuppressed. Commonly, antibody detection is performed based on traditional techniques, including CF and ID assays. Although ELISA and a lateral flow immunoassay (LFI) have been developed in an effort to improve the diagnostic accuracy and to improve the performance of laboratory tests, CF and ID continue to be widely used due to their simplicity and reliability [30]. Despite these advantages, ID tests can present great variability in terms of sensitivity [31], and cross-reactions with *Histoplasma* antigens can occur [32], even against purified antigens [23]. In addition, ID tests can have negative results at the beginning of the infection and in immunosuppressed patients.

Therefore, in the search for biomarkers for the diagnosis of coccidioidomycosis, our work identifies antigens of *Coccidioides* spp. by the recognition of sera from patients with coccidioidomycosis through the SDS-PAGE and western blot methods. In this study, coccidioidins were used because they are antigens containing proteins and polysaccharides. The coccidioidins are crude culture filtrates obtained from prolonged incubation and autolysis of the mycelial phase of the *Coccidioidess* spp. fungus in liquid media. On the other hand, the spherulin are extracts of the spherules of the fungus produced in vitro in a chemically defined liquid medium. The advantage of this antigen is that it is obtained from the parasitic phase of the fungus, which is in contact with the tissue of the host, while the mycelial phase of the fungus exists in the soil, so it is assumed that the antigens made from spherules would contain more relevant antigens in their participation of the host immune response; in addition, the spherulin is more sensitive than mycelial-based coccidioidin [33]. However, the utility of both preparations is recognized by clinicians, so in this study, we use the coccdidioidin, which is the antigen most commonly used in the diagnosis of this disease [26,34,35]. On the other hand, it has been shown that this antigen is considerably more specific than spherulin [36].

Of all the coccidioidins of both *C. immitis* and *C. posadasii*, no specific band was found for either species. However, all coccidioidins showed bands of 20, 25, 28, 50, 75, and 100 kDa; therefore, only one coccidioidin representative of each species was chosen (HU-1 for *C. posadasii* and M40-05 for *C. immitis)* to carry out the western blot assays with 27 serum samples from patients with proven coccidioidomycosis. Most serum samples recognized the 28, 37, 50, and 100 kDa bands, which were sequenced and analysed. A specific peptide was identified for *C. immitis* from the GPI anchored serine-threonine rich protein OS *Coccidioides immitis,* corresponding to the 100 kDa band, and another peptide corresponding to the “uncharacterized protein OS *Coccidioides immitis*”, which recognizes *C. immitis* and *C. posadasii*. Additionally, a peptide from the protein “fatty acid amide hydrolase 1 OS *Coccidioides posadasii*” was chosen, corresponding to the 50 kDa band, which is present in both *C. immitis* and *C. posadasii*. Importantly, the selected peptides were chosen based on the elimination of shared regions with closely related fungi, such as *H. capsulatum*, *Aspergillus* spp., *Blastomyces* spp., and *Paracoccidioides* spp. These are the fungi with which the selected peptides share the antigenic fractions responsible for cross-immunity [20].

In turn, the sequenced peptides of each band were also analysed by the BLAST protein program. Of the 353 peptides, only 35 showed 100% identity with sequences of *Coccidioides* spp. and an E-value <0.001, while the remainder shared sequences with related fungi (*Histoplasma, Blastomyces*, *Paracoccidioides*, *Aspergillus*, and *Criptococcus*). Of the selected peptides, one showed identity with only *C. immitis*, and another showed identity with only *C. posadasii*, so these peptides can be used for epidemiological studies because they discriminate between the two species. The low percentage found for species-specific peptides coincides with that reported by Mitchell et al. [37], who reported that both species of *Coccidioides* are closely related at the proteomic level. On the other hand, the rest of the peptides that were specific for the genus *Coccidioides* can represent an important tool for the diagnosis of coccidioidomycosis, with the advantage that they originate from antigenic fractions that are recognized by antibodies present in patients with coccidioidomycosis. In addition, peptides are now considered to be important; previously, an extensive area for the recognition of the antigen was assumed in the antibody-antigen interaction, where relatively large interaction sites are involved (>6 amino acids). For this reason, the biotechnology industry has only produced products with large recognition sites—either the complete ligand or receptor, or a substantial part of these. Today, it has been shown to be beneficial to use only one molecule representative of the binding site involved in the recognition event instead of an entire protein [29]. Such molecules, for example, peptides or mimotope peptides, with a size between six and 20 amino acids, have multiple advantages compared to the native protein in terms of production, storage, and safety. Moreover, very large protein molecules turn out to be pleiotropic; that is, they have more than one function. Often, these functions are found in separate sites of the molecule [38,39], and therefore, they can be separated using molecules that mimic the appropriate site. Thus, unwanted immune responses are less likely to occur with small peptides than with large proteins. Consequently, peptides can be used as diagnostic reagents, vaccines, and antifungals [29,40,41,42,43].

As a perspective of this study, the peptides selected as specific for *Coccidioides* spp., could be analyzed through immune-informatics and computational tools to find epitopes that are capable of inducing a humoral immune and cellular response and evaluating its usefulness in obtaining a vaccine or for diagnosis. In the case of reaching a diagnosis, this will be important, since the use of chemically defined antigens, such as peptides, can replace the classical immunological tests, which use undefined antigens that show variability in the sensitivity and specificity of the results [44,45].

## 4. Materials and Methods

### 4.1. Isolates

Eleven clinical isolates of *Coccidioides* spp. were used, consisting of nine isolates from Mexico and two isolates from Argentina. The clinical isolates were identified at the species level according to Duarte-Escalante et al. [46], using the method of Bialek et al. [47]. The isolates were preserved at 4 °C in vials with sterile water and in tubes with mycobiotic agar (Bioxón, México, MX), with and without mineral oil. All the procedures of this study were carried out in a class-3 biosecurity area. The isolates are found in the fungal collection of the Molecular Mycology Laboratory, Microbiology and Parasitology Department, School of Medicine, National Autonomous University of Mexico (*Universidad Nacional Autónoma de México—UNAM*). Their source and geographic origin are shown in Table 4.

### 4.2. Biological Samples

Twenty-seven serum samples from patients with proven coccidioidomycosis were used. The serum samples were provided by Dr. Carlos Cabello from the National Institute of Respiratory Diseases (*Instituto Nacional de Enfermedades Respiratorias—INER*) (Table 5).

### 4.3. Obtaining Coccidioidins

To demonstrate the antigenic identity of the study isolates, coccidioidins were obtained according to Smith et al. [48]. The mycelial phase of each isolate was inoculated in Smith’s medium (0.3 g of ferric citrate, 1.31 g of potassium dibasic phosphate, 0.4 g of anhydrous sodium citrate, 1.5 g of magnesium sulphate, 10 g of glucose, 25.6 mL of glycerine, 7 g of asparagine, 7 g of ammonium chloride, brought to 1000 mL) and incubated at 28 °C for three months. Next, each culture was inactivated with thimerosal (1:5000) for 20 days, and subsequently, the mycelial mass was separated by filtration. Each culture’s filtrate was concentrated 100 times using the Amicon system (Sigma-Aldrich, St. Louis, MO, USA) and a 10 kDa exclusion membrane (Millipore Corporation, Burlington, MA, USA). The concentrated filtrate was dialyzed against deionized water for 24 h, with several changes, to finally obtain concentrated exoantigens, known as crude antigens (coccidioidins). The antigenic identification was carried out by the ID immune method, as described by Ouchterlony and Nilsson [49]. Positive and negative sera were used, as well as a reference antigen. The immunological identification of coccidioidins was carried out based on the appearance of identity bands. For subsequent trials, the protein concentration of each antigenic extract was determined by the method of Bradford et al. [50], using bovine serum albumin (BSA) as a control.

### 4.4. Sodium Dodecyl Sulfate-Polyacrylamide Gel Electrophoresis (SDS-PAGE)

To determine the components and molecular weights of the different coccidioidins, one-dimensional electrophoresis was carried out under denaturing conditions using the batch method [51], as described below. The coccidioidins were adjusted to a concentration of 80 µg/mL with 0.5 M Tris-HCl buffer pH 6.8 and 0.1% SDS, and the samples were heated at 100 °C for 5 min. Next, the coccidioidins were run on gels with different acrylamide concentrations (7.5%, 10%, and 12%) in buffer containing 0.025 M Tris-HCl, 0.2 M glycine, and 1% SDS. A molecular size marker of 250–15 kDa was used (Bio-Rad Laboratories Inc., Hercules, CA, USA). Electrophoresis was carried out with a Bio-Rad vertical electrophoresis system, with a constant current of 20 mA for 2 h. The gels were stained with Coomassie blue R250 (Sigma-Aldrich, St. Louis, MO, USA). A binary data matrix for the presence/absence of bands was developed based on the analysis with the Bio-Rad imaging system of the electrophoretic patterns obtained with the analysed exoantigens to identify, with greater precision, the existence of species-specific bands.

### 4.5. Recognition of Antigenic Fractions of Coccidioidins by Western Blot (WB)

The WB assays were carried out according to the method described by Towbin et al. [52]. The SDS-PAGE gels were placed on 0.45 µm pore nitrocellulose membranes (Bio-Rad, Hercules, CA, USA). The transfer of the antigens to the membranes was carried out in a Mini Trans-Blot Cell device (Bio-Rad, Hercules, CA, USA) at 4 °C, with a constant voltage of 100 V for 2 h using transfer buffer (0.025 M Tris-base pH 8.3, 0.192 M glycine, 0.37 g of SDS and 20% methanol in one litre of deionized water). After the transfer, the nitrocellulose membrane was washed three times for 5 min with TBS (50 mM Tris, 150 mM NaCl, 0.1% Tween-20). Free reactive sites were blocked overnight with 5.0% BSA in TBS/Tween at 4 °C with occasional shaking. The membranes were washed with deionized water and with TBS-Tween three times, 10 min per wash. The test serum was added (previously diluted (1/250) with TBS-Tween) and incubated at room temperature for 2 h. The membrane was washed three times with TBS-Tween for 5 min. Next, peroxidase-conjugated anti-human gamma globulin was added (Sigma-Aldrich, St. Louis, MO, USA) at a dilution of 1/5000 and incubated for 1 h at room temperature, followed by washing with TBS for 5 min. Subsequently, we added 6 mL of Luminata Crescendo substrate (Merck, Kenilworth, NJ, USA) and incubated the sample for 2–5 min. Finally, to develop the sample, the membrane was exposed to a Kodak X-OMAT AR FILM radiography plate (10 × 10 cm). A presence/absence matrix was built to identify bands recognized by sera from patients with proven coccidioidomycosis and to select those that presented the highest recognition frequency with the tested sera.

### 4.6. Protein Identification of the Antigenic Fractions of C. immitis and C. posadasii by Mass Spectrometry

Differential bands were selected for identification by mass spectrometry. The sample treatment prior to identification was carried out according to the protocol developed in the Research and Industry Support Services Unit (*Unidad de Servicios de Apoyo a la Investigación y la Industria- USAII*) of the School of Chemistry at the National Autonomous University of Mexico. Briefly, the gel bands were cut into small fragments and destained with 50% methanol (*v*/*v*) and 5% acetic acid (*v*/*v*) for 12 h. They were then washed with distilled water and incubated for 15 min (twice) in 100 mM ammonium bicarbonate. They were reduced in 50 mM dithiothreitol (DTT) for 45 min and alkylated with 30 mM iodoacetamide for 2 h. Subsequently, they were washed three times with 100 mM ammonium bicarbonate. The gel samples were then completely dehydrated with 100% acetonitrile and brought to full dryness. The in-gel digestion was performed with 30 μL of porcine trypsin (Sigma-Aldrich, St. Louis, MO, USA) modified from a solution containing 20 ng/μL and incubated for 18 h at 37 °C.

The peptides were extracted by sonication in 50% acetonitrile (*v*/*v*) and 5% formic acid (*v*/*v*) and brought to full dryness. Subsequently, they were resuspended in 20 μL of 1% formic acid (*v*/*v*), desalted on Ziptip C18 columns, and eluted in 12 μL of mobile phase (97% water, 3% acetonitrile, and 0.1% formic acid).

Peptide analysis by mass spectrometry was carried out using an integrated high-definition SYNAPT G2 nano-LC-ESI-MS/MS system (Waters Corporation, Milford, MA, USA) equipped with a NanoLockSpray ion source and coupled in line to a nanoACQUITY Ultra Performance Liquid Chromatograph (UPLC, Waters Corporation, Milford, MA, USA). The binary solvent system was 2% acetonitrile in Milli-Q water with 0.1% formic acid (mobile phase A) and 98% acetonitrile in Milli-Q water with 0.1% formic acid (mobile phase B). The sample was injected into a C18 UPLC trap column (5 μm, 180 μmx, and 20 mm; Waters Corporation, Milford, MA, USA) and washed with 100% mobile phase A at a flow rate of 15 μL/min. The peptides were separated on a BEH, C18 UPLC column (1.7 μm, 75 μm and 100 mm; Waters Corporation, Milford, MA, USA) using a linear gradient up to 40% of mobile phase B with a flow rate of 0.3 μL/min.

We searched the UNIPROT database (https://www.uniprot.org) using trypsin as a specific protease. Peptides with a Protein Lynx Global Server (PLGS) score of >95% confidence were accepted as correct. The scoring threshold under the previous conditions was 52 for *p* < 0.05.

### 4.7. Analysis of Assembled Sequences

Assembled sequences corresponding to the bands at 100, 50, 37, and 28 kDa were analysed by the BLAST protein program (Basic Local Alignment Search Tool) [53] (www.blast.ncbi.nlm.nih.gov/blast.cgi) to choose sequences that showed an identity and similarity of 100% with *Coccidioides* spp. and an identity <50% with related fungi. Subsequently, the selected sequences were aligned with the sequences that presented <50% identity to select a fragment that was not shared with the related fungi. The alignment was carried out with the UNIPROT program (https://www.uniprot.org/align).

### 4.8. Peptide Analysis

All of the peptides from each assembled protein identified in the sequencing were analysed through the BLAST protein program. The identity, similarity, and e-value parameters were considered for analysis. Peptides that had 100% identity and similarity with *C. immitis* and *C. posadasii* and a *p*-value < 0.001 were selected.

## 5. Conclusions

The peptides identified in this work can be an important tool for the diagnosis of coccidioidomycosis since they arise from antigenic fractions that are recognized by antibodies present in the serum of patients with coccidioidomycosis. In addition, the identification of specific-species peptides can be useful for the epidemiological study of this disease.

## Figures and Tables

**Table 1:** Matrix of presence/absence of bands in the electrophoretic patterns of coccidioidins of *C. immitis* and *C. posadasii.*

Coccidioidins											
kDa	QR	HU-1	GGM	ZVJ	073329	083376	M40-05	PRA	30.1	43.3	62
100	1	1	1	1	1	0	1	0	1	1	0
75	1	1	1	1	1	1	1	1	1	1	1
65	1	1	1	1	1	0	1	1	1	1	0
55	1	1	1	1	1	1	1	1	1	1	1
50	1	1	1	1	1	1	1	1	1	1	1
37	1	1	1	1	1	0	1	1	0	0	
35	0	1	1	1	1	1	1	1	1	1	1
30	1	1	1	1	1	1	1	1	1	1	1
28	1	1	1	1	1	1	1	1	1	1	1
23	0	1	1	1	1	1	0	0	0	0	1
21	0	1	0	0	0	1	0	0	0	0	1
20	1	1	1	1	1	1	1	1	1	1	1
19	1	1	1	0	0	1	0	1	0	0	0
18	0	0	1	1	1	1	1	1	1	1	0
16	1	1	1	1	1	1	1	0	1	1	1

**Table 2:** Recognition frequency of anti-*Coccidioides* sera with *Coccidioides* spp. antigens.

kDa	Frequency (%) Anti-*C. immitis*	Frequency (%) Anti-*C. posadasii*
250	21.42 (6/28)	10.71 (3/28)
150	10.71 (3/28)	10.71 (3/28)
100	46.42 (13/28)	46.42 (13/28)
75	10.71 (3/28)	10.71 (3/28)
50	42.85 (12/28)	42.85 (12/28)
37	28.57 (8/28)	28.57 (8/28)
28	20.0 (5/28)	25.0 (7/28)

**Table 3:** Identification of proteins that recognize *Coccidioides* spp.

Access Number	Description	Alignment	Query Cover	E-Value	Identity
**100 kDa band**					
A0A0J6YAG6_COCIT	GPI anchored serine threonine-rich protein OS *C. immitis*	*C. immitis*	93%	7 × 10^−96^	75%
		*C. posadasii*	93%	1 × 10^−94^	74%
		*H. capsulatum*	68%	8 × 10^−38^	52%
		*A. fumigatus*	63%	6 × 10^−45^	57%
		*P. brasiliensis*	58%	2 × 10^−30^	49%
A0A0J8R1I4_COCIT	Uncharacterized protein OS *C. immitis*	*C. immitis*	100%	0	90%
		*C. posadasii*	100%	0	89%
		*P. lutzi*	49%	1 × 10^−67^	60%
**50 kDa band**		*H. capsulatum*	47%	1 × 10^−64^	58%
A0A0J6F383_COCPO	Fatty acid amide hydrolase 1 OS *C. posadasii*	*C. posadasii*	100%	0	97%
		*C. immitis*	100%	0	96%
		*P. brasiliensis*	91%	0	54%
		*P. lutzi*	91%	0	54%
		*H. capsulatum*	88%	0	55%

**Table 4:** Source and geographical origin of *Coccidioides* spp. isolates.

Isolate	Specie	Source	Geographical Origin
HU1	*C. posadasii*	ND	Monterrey, MX
073129	*C. posadasii*	Biopsy	Salta, AR
083376	*C. posadasii*	BAL	Catamarca, AR
30.1	*C. immitis*	Sputum	Tijuana, MX
43.3	*C. immitis*	Sputum	Guerrero, MX
62	*C. immitis*	Sputum	ND
GGM	*C. posadasii*	Sputum	Baja California, MX
ZVJ	*C. posadasii*	Pulmonary nodule	Michoacán, MX
QR	*C. posadasii*	Knee puncture	ND
PRA	*C. immitis*	BAL	Tijuana, MX
M40-05	*C. immitis*	Wound secretion	Guadalajara, MX

BAL: bronchoalveolar lavage.

**Table 5:** Serum samples from patients with acute pulmonary or disseminated coccidioidomycosis.

Sera	Gender	Age	IDG	PTC	ELISA	Culture
1	F	31	Positive	1:32	ND	Positive
2	F	39	Negative	1:32	ND	Negative
3	M	75	Positive	1:16	1:256	Positive
5	F	53	Positive	1:16	ND	Negative
9	F	37	Negative	1:32	ND	Negative
10	F	38	Negative	1:2	ND	Negative
14	M	41	Positive	1:8	ND	Negative
17	F	38	Negative	1:16	ND	Negative
18	F	46	Negative	1:64	ND	Negative
23	M	79	Negative	1:32	ND	Negative
29	M	42	Positive	1:32	1:128	Positive
31	M	19	Positive	1:128	ND	Positive
32	M	32	Positive	1:2	1:256	Positive
35	M	44	Positive	1:2	ND	Positive
45	F	47	Negative	1:8	ND	Negative
52	M	42	Negative	1:256	ND	Negative
53	F	19	Negative	1:64	ND	Negative
55	M	45	Positive	1:32	ND	Negative
59	M	11	Negative	1:16	ND	Negative
61	F	26	Negative	Negative	ND	Negative
64	F	63	Positive	Negative	1:128	Negative
69	M	35	Negative	1:8	ND	Positive
74	F	35	Negative	1:8	ND	Negative
75	F	57	Positive	1:64	ND	Negative
81	M	45	Positive	1:32	1:256	Positive
82	M	67	Negative	1:64	ND	Negative
85	F	77	Positive	1:64	ND	Negative

## Supplementary Materials

Supplementary data associated with this article can be found in the online version.
