# Supplementary material for: Selection of Specific Peptides for Coccidioides spp. Obtained from Antigenic Fractions through SDS-PAGE and Western Blot Methods by the Recognition of Sera from Patients with Coccidioidomycosis
Source: Molecules. 2018 Nov 30;23(12):3145. doi: 10.3390/molecules23123145 (PMC6321320; doi:10.3390/molecules23123145)
Supplement: Supplementary file 1 [file molecules-23-03145-s001.zip › molecules-386218-submit-supplementary-original/Tables S2-S5.pdf]

Supplementary

Selection of Specific Peptides for *Coccidioides* spp. Obtained from Antigenic Fractions through SDS-PAGE and Western Blot Methods by the Recognition of Sera from Patients with Coccidioidomycosis

Esperanza Duarte Escalante [1], María Guadalupe Frías De León [2], Luz Gisela Martínez García [1], Jorge Herrera [3], Gustavo Acosta Altamirano [2], Carlos Cabello [4], Gabriel Palma [4], María del Rocío Reyes Montes [1,*]

Table S2. Peptides of *Coccidioides* spp. corresponding to the 100 kDa band.

Peptide	Specie	Accession
ISVSNIVVDQCSK	proline-rich antigen [*Coccidioides posadasii* C735 delta SOWgp]	XP_003069153.1
	proline-rich antigen [*Coccidioides immitis* RS]	XP_001240075.1
	proline rich antigen 2 [*Coccidioides immitis* RS]	XP_001239939.1
	proline rich antigen 2 [*Coccidioides posadasii* C735 delta SOWgp],	XP_003067448.1
		AFL65837.1
	proline-rich-antigen, partial [*Coccidioides posadasii*],	AFL65836.1
	proline-rich-antigen, partial [*Coccidioides posadasii*],	AFR78204.1
	proline-rich antigen, partial [*Coccidioides immitis*],	AFR78201.1
	proline-rich antigen, partial [*Coccidioides posadasii*],	AFL65860.1
	proline-rich antigen, partial [*Coccidioides posadasii*],	AAP84614.1
	Prp 2 CRoW domain *Coccidioides posadasii*,	AAP84613.1
	Ag2/Pra CRoW domain	
NIIYVATLGSSPSIR	secreted dipeptidyl peptidase [*Coccidioides posadasii* str. Silveira],	EFW16303.1
		KMM67819.1
	dipeptidyl-peptidase 5 [*Coccidioides posadasii* RMSCC 3488],	XP_003070019.1
	Dipeptidyl-peptidase V precursor, putative [*Coccidioides*	XP_001242886.1

	posadasii C735 delta SOWgp],<br>secreted dipeptidyl peptidase [*Coccidioides immitis* RS],<br>dipeptidyl-peptidase V [*Coccidioides immitis* RMSCC 2394],<br>dipeptidyl-peptidase V [*Coccidioides immitis* H538.4]	KMP03935.1<br>KMU86093.1
VLVTGTSLWSNTLYYTTGPR	secreted dipeptidyl peptidase [*Coccidioides posadasii* str. Silveira],<br>dipeptidyl-peptidase 5 [*Coccidioides posadasii* RMSCC 3488],<br>Dipeptidyl-peptidase V precursor, putative [*Coccidioides posadasii* C735 delta SOWgp],<br>secreted dipeptidyl peptidase [*Coccidioides immitis* RS],<br>dipeptidyl-peptidase V [*Coccidioides immitis* RMSCC 2394],<br>dipeptidyl-peptidase V [*Coccidioides immitis* H538.4]	EFW16303.1<br>KMM67819.1<br>XP_003070019.1<br>XP_001242886.1<br>KMP03935.1<br>KMU86093.1
NVGSVSSYSFLGR	secreted dipeptidyl peptidase [*Coccidioides posadasii* str. Silveira],<br>dipeptidyl-peptidase 5 [*Coccidioides posadasii* RMSCC 3488],<br>Dipeptidyl-peptidase V precursor, putative [*Coccidioides posadasii* C735 delta SOWgp],<br>secreted dipeptidyl peptidase [*Coccidioides immitis* RS],<br>dipeptidyl-peptidase V [*Coccidioides immitis* RMSCC 2394],<br>dipeptidyl-peptidase V [*Coccidioides immitis* H538.4]	EFW16303.1<br>KMM67819.1<br>XP_003070019.1<br>XP_001242886.1<br>KMP03935.1<br>KMU86093.1
QEVQAWIVKPENFDPNKK	secreted dipeptidyl peptidase [*Coccidioides posadasii* str. Silveira],<br>dipeptidyl-peptidase 5 [*Coccidioides posadasii* RMSCC 3488],<br>Dipeptidyl-peptidase V precursor, putative [*Coccidioides posadasii* C735 delta SOWgp],<br>secreted dipeptidyl peptidase [*Coccidioides immitis* RS],<br>dipeptidyl-peptidase V [*Coccidioides immitis* H538.4],<br>d ipeptidyl-peptidase V [*Coccidioides immitis* RMSCC 2394]	EFW16303.1<br>KMM67819.1<br>XP_003070019.1<br>XP_001242886.1<br>KMU86093.1<br>KMP03935.1
NIIYVATLGSSPSIR	secreted dipeptidyl peptidase [*Coccidioides posadasii* str.	EFW16303.1

	Silveira],<br>dipeptidyl-peptidase 5 [*Coccidioides posadasii* RMSCC 3488],<br>Dipeptidyl-peptidase V precursor, putative [*Coccidioides posadasii* C735 delta SOWgp],<br>secreted dipeptidyl peptidase [*Coccidioides immitis* RS],<br>dipeptidyl-peptidase V [*Coccidioides immitis* RMSCC 2394],<br>dipeptidyl-peptidase V [*Coccidioides immitis* H538.4],	KMM67819.1<br>XP_003070019.1<br>XP_001242886.1<br>KMP03935.1<br>KMU86093.1
QEVQAWIVKPENFDPNKK	secreted dipeptidyl peptidase [*Coccidioides posadasii* str. Silveira],<br>dipeptidyl-peptidase 5 [*Coccidioides posadasii* RMSCC 3488],<br>Dipeptidyl-peptidase V precursor, putative [*Coccidioides posadasii* C735 delta SOWgp],<br>secreted dipeptidyl peptidase [*Coccidioides immitis* RS],<br>dipeptidyl-peptidase V [*Coccidioides immitis* H538.4],<br>dipeptidyl-peptidase V [*Coccidioides immitis* RMSCC 2394]	EFW16303.1<br>KMM67819.1<br>XP_003070019.1<br>XP_001242886.1<br>KMU86093.1<br>KMP03935.1
TPQLVIHNDLDFR	secreted dipeptidyl peptidase [*Coccidioides posadasii* str. Silveira],<br>dipeptidyl-peptidase 5 [*Coccidioides posadasii* RMSCC 3488],<br>Dipeptidyl-peptidase V precursor, putative [*Coccidioides posadasii* C735 delta SOWgp],<br>secreted dipeptidyl peptidase [*Coccidioides immitis* RS],<br>dipeptidyl-peptidase V [*Coccidioides immitis* RMSCC 2394]	EFW16303.1<br>KMM67819.1<br>XP_003070019.1<br>XP_001242886.1<br>KMP03935.1
NGLDVFLNIPYGQDTSGEHR	lipase, putative [*Coccidioides posadasii* C735 delta SOWgp],<br>acetylcholinesterase [*Coccidioides posadasii* str. Silveira],<br>hypothetical protein CIMG_00118 [*Coccidioides immitis* RS],<br>carboxylesterase 2 [*Coccidioides immitis* RMSCC 3703],<br>carboxylesterase 2 [*Coccidioides immitis* H538.4]	XP_003066793.1<br>EFW18154.1<br>XP_001246347.2<br>KMU75838.1<br>KMU88343.1

Table S3. Peptides of *Coccidioides* spp. corresponding to the 50 kDa band.

Peptide	Specie	Accession
DAITLANHAK	CHA1 [*Coccidioides posadasii*]	XP_003070371.1
PSGEVIGNLDR	CHA1 [*Coccidioides posadasii*,<br>hypothetical protein CIMG_08329 [*Coccidioides immitis*],	XP_003070371.1<br>XP_001241166.1
ETNVDGIIGPVAVPAAAR	acetamidase [*Coccidioides immitis*,<br>acetamidase, putative [*Coccidioides posadasii*,<br>hypothetical protein CISG_07260 [*Coccidioides immitis*]	KMP01873.1<br>XP_003065414.1<br>XP_001248096.1<br>KMU79093.1
NIIYVATLGSSPSIR	secreted dipeptidyl peptidase [*Coccidioides posadasii*, *C. immitis*],<br>dipeptidyl-peptidase 5 [*Coccidioides posadasii*, *C immitis*]	EFW16303.1<br>KMM67819.1<br>XP_003070019.1<br>XP_001242886.1<br>KMP03935.1<br>KMU86093.1
NEWDVIDLESSK	secreted dipeptidyl peptidase [*Coccidioides posadasii*, *C. immitis*],<br>dipeptidyl-peptidase 5 [*Coccidioides posadasii*, *C. immitis*]	EFW16303.1<br>KMM67819.1<br>XP_003070019.1<br>XP_001242886.1<br>KMP03935.1<br>KMU86093.1

Table S4. Peptides of *Coccidioides* spp. corresponding to the 37 kDa band.

Peptide	Specie	Accesion
NIVITDYSTGK	hypothetical protein CPAG_01767 [*Coccidioides posadasii* RMSCC	KMM65416.1
	3488]	XP_003066044.1
	Glycosyl hydrolase family 16 protein [*Coccidioides posadasii* C735 delta	XP_001247331.1
	SOWgp]	KMU72732.1
	cell wall glucanase [*Coccidioides immitis* RS]	EFW21954.1
	hypothetical protein CISG_03166 [*Coccidioides immitis* RMSCC 3703]	KMU83540.1
	conserved hypothetical protein [*Coccidioides posadasii* str. Silveira]	
	ice nucleation protein [*Coccidioides immitis* H538.4]	
ETNVDGIIGPVAVPAAAR	acetamidase [*Coccidioides immitis* RMSCC 2394],	KMP01873.1
	acetamidase, putative [*Coccidioides posadasii* C735 delta SOWgp],	XP_003065414.1
	acetamidase [*Coccidioides immitis* RS],	XP_001248096.1
	hypothetical protein CISG_07260 [*Coccidioides immitis* RMSCC 3703]	KMU79094.1
LPADQAINNADTFAIYAADSYR	metalloproteinase 4 [*Coccidioides immitis* RS]	XP_001246737.2
EASKDDANVSVQLSAVGNTMVK	neutral protease 2 precursor, putative [*Coccidioides posadasii* C735 delta	XP_003066497.1
	SOWgp], metalloproteinase 4 [*Coccidioides posadasii*],	
	neutral protease 2 [*Coccidioides immitis* RMSCC 3703],	
	neutral protease 2 [*Coccidioides immitis* H538.4],	
	neutral protease 2 [*Coccidioides posadasii* RMSCC 3488],	
	metalloproteinase 4 [*Coccidioides immitis* RS]	
LPADQAINNADTFA	neutral protease 2 precursor, putative [*Coccidioides posadasii* C735 delta	XP_003066497.1
	SOWgp],	AAY45754.1
	metalloproteinase 4 [*Coccidioides posadasii*],	KMU76812.1
	neutral protease 2 [*Coccidioides immitis* RMSCC 3703],	KMU84566.1
	neutral protease 2 [*Coccidioides immitis* H538.4],	KMM66042.1

	neutral protease 2 [*Coccidioides posadasii* RMSCC 3488],<br>metalloproteinase 4 [*Coccidioides immitis* RS]	XP_001246737.2
LPADQAINNADTFAIYAA	neutral protease 2 precursor putative [*Coccidioides posadasii* C735 delta SOWgp], metalloproteinase 4 [*Coccidioides posadasii*],<br>neutral protease 2 [*Coccidioides immitis* RMSCC 3703],<br>neutral protease 2 [*Coccidioides immitis* H538.4],<br>neutral protease 2 [*Coccidioides posadasii* RMSCC 3488],<br>metalloproteinase 4 [*Coccidioides immitis* RS]	XP_003066497.1<br>AAY45754.1<br>KMU76812.1<br>KMU84566.1<br>KMM66042.1<br>XP_001246737.2
QYISVISPTYNLIANR	wall-associated proteinase [*Coccidioides posadasii* str. Silveira],<br>serine proteinase [*Coccidioides posadasii*],<br>wall-associated proteinase [*Coccidioides posadasii* RMSCC 3488],<br>wall-associated proteinase precursor [*Coccidioides posadasii* C735 delta SOWgp],<br>wall-associated proteinase [*Coccidioides immitis* RS],<br>wall-associated proteinase [*Coccidioides immitis* RMSCC 3703]	EFW19467.1<br>CAA44828.1<br>KMM71928.1<br>XP_003067238.1<br>XP_001239683.1<br>KMU74068.1
VTDNEIHWYANQVVEGAPPMK	serine proteinase [*Coccidioides posadasii*],<br>Wall-associated proteinase precursor [*Coccidioides posadasii* C735 delta SOWgp],<br>wall-associated proteinase [*Coccidioides immitis* RS]	CAA44828.1
AVDPEITEPFR	FAD-dependent oxygenase [*Coccidioides posadasii* str. Silveira],<br>FAD-dependent oxygenase [*Coccidioides immitis* RS],<br>hypothetical protein CIRG_05419 [*Coccidioides immitis* RMSCC 2394],<br>FAD binding domain containing protein [*Coccidioides posadasii* C735 delta SOWgp], hypothetical protein CIHG_04911 [*Coccidioides immitis* H538.4]	EFW17327.1<br>XP_001246156.2<br>KMP05738.1<br>XP_003067765.1<br>KMU86971.1

Table S5. Peptides of *Coccidioides* spp. corresponding to the 28 kDa band.

Peptide	Specie	Accesion
ATLPDLPYDYGALE	*C. posadasii* (superoxide dismutase 2),<br>*C. immitis* (superoxide dismutase)	KMM64900.1<br>XP_001247816.1
PDLPYDYGALEPSISGR	superoxide dismutase 2 [*Coccidioides posadasii* RMSCC 3488],<br>superoxide dismutase [*Coccidioides immitis* RS]	KMM64900.1<br>XP_001247816.1
DDKFGSLEEFK	superoxide dismutase 2 [*Coccidioides posadasii* RMSCC 3488]	KMM64900.1
AFDETNFYFDPK	hypothetical protein CIMG_00536 [*Coccidioides immitis* RS],<br>hypothetical protein CPC735_056990 [*Coccidioides posadasii* C735 delta SOWgp],<br>hypothetical protein CISG_05614 [*Coccidioides immitis* RMSCC 3703]	XP_001246765.2<br>XP_003066474.1<br>KMU76781.1
IPLALFNVDRPR	hypothetical protein CIMG_00536 [*Coccidioides immitis* RS],<br>hypothetical protein CPC735_056990 [*Coccidioides posadasii* C735 delta SOWgp]	XP_001246765.2<br>XP_003066474.1
PLALFNVDRPR	hypothetical protein CIMG_00536 [*Coccidioides immitis* RS],<br>hypothetical protein CPC735_056990 [*Coccidioides posadasii* C735 delta SOWgp]	XP_001246765.2<br>XP_003066474.1
LALFNVDRPR	hypothetical protein CIMG_00536 [*Coccidioides immitis* RS],<br>hypothetical protein CPC735_056990 [*Coccidioides posadasii* C735 delta SOWgp]	XP_001246765.2<br>XP_003066474.1
PALSGTATFDLK	hypothetical protein CPAG_01767 [*Coccidioides posadasii* RMSCC 3488],<br>Glycosyl hydrolase family 16 protein [*Coccidioides posadasii* C735 delta SOWgp],<br>cell wall glucanase [*Coccidioides immitis* RS],<br>conserved hypothetical protein [*Coccidioides posadasii* str. Silveira]	KMM65416.1<br>XP_003066044.1<br>XP_001247331.1<br>EFW21954.1
TVTPNDGNAR	hypothetical protein CPAG_01767 [*Coccidioides posadasii* RMSCC 3488],<br>Glycosyl hydrolase family 16 protein [*Coccidioides posadasii* C735 delta SOWgp],	KMM65416.1<br>XP_003066044.1

	cell wall glucanase [*Coccidioides immitis* RS],	XP_001247331.1
	hypothetical protein CISG_03166 [*Coccidioides immitis* RMSCC 3703],	KMU72732.1
	conserved hypothetical protein [*Coccidioides posadasii* str. Silveira]	EFW21954.1
VTYEEVASSLCGLK	allergen Asp f 15 [*Coccidioides immitis* RMSCC 3703],	KMU74303.1
	CS antigen [*Coccidioides posadasii* C735 delta SOWgp],	XP_003065978.1
	heat-stable antigen [*Coccidioides immitis* RS]	XP_001247410.1
TIFLTAIDHSNSGFNIAK	19 kDa antigen [*Coccidioides posadasii*],	AAB00101.1
	allergen Asp f 15 [*Coccidioides immitis* RMSCC 3703],	KMU74303.1
	CS antigen [*Coccidioides posadasii* C735 delta SOWgp],	XP_003065978.1
	heat-stable antigen [*Coccidioides immitis* RS]	XP_001247410.1
FLTAIDHSNSGFNIAK	19 kDa antigen [*Coccidioides posadasii*],	AAB00101.1
	allergen Asp f 15 [*Coccidioides immiti*s RMSCC 3703],	KMU74303.1
	CS antigen [*Coccidioides posadasii* C735 delta SOWgp],	XP_003065978.1
	heat-stable antigen [*Coccidioides immitis* RS]	XP_001247410.1
EIALWFSEGELIK	nucleoside diphosphate kinase [*Coccidioides immitis* RS]	XP_001242666.1
IVQSDAATLVR	acetamidase [*Coccidioides immitis* RMSCC 2394],	KMP01873.1
	acetamidase, putative [Coccidioides posadasii C735 delta SOWgp],	XP_003065414.1
	acetamidase [*Coccidioides immitis* RS]	XP_001248096.1
VYFDISIGNR	peptidyl-prolyl cis-trans isomerase D [*Coccidioides immitis* H538.4],	KMU86947.1
	41 kDa peptidyl-prolyl cis-trans isomerase, putative [*Coccidioides posadas*ii C735 delta	XP_003067748.1
	SOWgp], peptidyl-prolyl cis-trans isomerase [*Coccidioides immitis* RS]	XP_001246138.1
